# Association between polymorphisms in the aryl hydrocarbon receptor repressor gene and disseminated testicular germ cell cancer

**DOI:** 10.3389/fendo.2013.00004

**Published:** 2013-02-14

**Authors:** Leon J. S. Brokken, Yvonne Lundberg-Giwercman, Ewa Rajpert-De Meyts, Jakob Eberhard, Olof Ståhl, Gabriella Cohn-Cedermark, Gedske Daugaard, Stefan Arver, Aleksander Giwercman

**Affiliations:** ^1^Department of Molecular Reproductive Medicine, Lund University Malmö, Sweden; ^2^Department of Growth and Reproduction, RigshospitaletCopenhagen, Denmark; ^3^Department of Oncology, Skåne University HospitalLund, Sweden; ^4^Department of Oncology–Pathology, Radiumhemmet, Karolinska Institute and University HospitalStockholm, Sweden; ^5^Department of Oncology, RigshospitaletCopenhagen, Denmark; ^6^Centre for Andrology and Sexual Medicine, Karolinska University Hospital Huddinge, Department of MedicineStockholm, Sweden; ^7^Reproductive Medicine Centre, Skåne University HospitalMalmö, Sweden

**Keywords:** aryl hydrocarbon receptor, aryl hydrocarbon receptor repressor, testicular germ cell cancers, genetic polymorphisms, genetic variation, association studies, metastasis, histology

## Abstract

In the Western world, testicular germ cell cancer (TGCC) is the most common malignancy of young men. The malignant transformation of germ cells is thought to be caused by developmental and hormonal disturbances, probably related to environmental and lifestyle factors because of rapidly increasing incidence of TGCC in some countries. Additionally, there is a strong genetic component that affects susceptibility. However, genetic polymorphisms that have been identified so far only partially explain the risk of TGCC. Many of the persistent environmental pollutants act through the aryl hydrocarbon receptor (AHR). AHR signaling pathway is known to interfere with reproductive hormone signaling, which is supposed to play a role in the pathogenesis and invasive progression of TGCC. The aim of the present study was to identify whether AHR-related polymorphisms were associated with risk as well as histological and clinical features of TGCC in 367 patients and 537 controls. Haplotype-tagging single-nucleotide polymorphisms (SNPs) were genotyped in genes encoding AHR and AHR repressor (AHRR). Binary logistic regression was used to calculate the risk of TGCC, non-seminoma versus seminoma, and metastasis versus localized disease. Four SNPs in *AHRR* demonstrated a significant allele association with risk to develop metastases (rs2466287: OR = 0.43, 95% CI 0.21–0.90; rs2672725: OR = 0.49, 95% CI: 0.25–0.94; rs6879758: OR = 0.27, 95% CI: 0.08–0.92; rs6896163: OR = 0.34, 95% CI: 0.12–0.98). This finding supports the hypothesis that compounds acting through AHR may play a role in the invasive progression of TGCC, either directly or through modification of reproductive hormone action.

## INTRODUCTION

Testicular germ cell cancer (TGCC) represents the most-frequently diagnosed cancer in young men between 20 and 40 years of age. The rates vary considerably according to region and ethnicity, which suggests that both environmental and genetic factors contribute to TGCC. In Europe, a three- to fourfold increased incidence has been noted during the past 30–40 years, which suggests that an increased exposure to persistent environmental pollutants may play an important role.

Common persistent environmental pollutants include dioxins and polycyclic aromatic hydrocarbons, which exert their toxic and carcinogenic effects through the ligand-activated transcription factor aryl hydrocarbon receptor (AHR). In complex with its binding partner AHR nuclear translocator (ARNT), it mediates cellular responses to xenobiotic compounds. The AHR/ARNT heterodimer stimulates the expression of AHR repressor (AHRR), which provides a negative feedback regulation by competing with ARNT for binding to AHR (Mimura et al., 1999).

Testicular germ cell cancer derives from carcinoma *in situ* (CIS) cells, also known as intratubular germ-cell neoplasia unclassified (Skakkebaek et al., 1982; Oosterhuis and Looijenga, 2005), that are believed to arise from primordial germ cells that are blocked in differentiation. The initial malignant transformation of germ cells is thought to be caused by hormonal disturbances in the microenvironment of differentiating germ cells (Skakkebaek et al., 1987; Sharpe and Skakkebaek, 1993; Rajpert-De Meyts et al., 1998). An association of TGCC with maternal estrogen and androgen levels in early pregnancy has recently been reported, supporting this hypothesis (Holl et al., 2009). In addition to altering transcription of steroidogenic genes, such as cytochrome P450 enzymes, the AHR signaling pathway has been shown to interface with estrogen and androgen receptor signaling (Jana et al., 1999; Kizu et al., 2003; Safe and Wormke, 2003; Boverhof et al., 2006; Beischlag et al., 2008; Kollara and Brown, 2010). Additionally, AHR plays a significant role in tumor promotion and progression by deregulation of cell–cell contact and inhibition of apoptosis (Mulero-Navarro et al., 2005; Diry et al., 2006; Carvajal-Gonzalez et al., 2009; Chopra et al., 2009; Dietrich and Kaina, 2010).

The risk of environment-related carcinogenesis depends not only on the magnitude of the exposure, but also on individual susceptibility to the pollutant. Therefore, we aimed to analyze the association between polymorphic variants of genes encoding AHR and AHRR and the risk as well as histological and clinical features of TGCC.

## MATERIALS AND METHODS

The cohort consisted of TGCC patients from Sweden and Denmark, two neighboring Scandinavian countries, with populations sharing largely the same genetic background as both descended from a common ancestral population.

### SWEDISH TGCC PATIENTS

All TGCC patients that were referred to the Department of Oncology, Skåne University Hospital, Lund, the Department of Oncology, Radiumhemmet or Södersjukhuset, Karolinska University Hospital, Stockholm since March 1996 and November 1998 until October 2006, respectively, were asked to participate in a study focusing on reproductive function. In total 460 patients were eligible for the study, of which 75 declined to participate and 45 were excluded due to linguistic problems, bilateral testicular cancer, physical handicap, or moving to another region. Seven patients were excluded due to compromising mental conditions, 10 were excluded due to contra-lateral testicular cancer diagnosed after inclusion and 3 died of progressive disease before blood samples were obtained. Among the 320 participants in the study on reproductive function, no DNA was available for 39. Additionally, 3 patients were excluded due to extra-gonadal germ cell cancer, leaving the total at 278. All patients gave their informed written consent and the study was approved by the Ethical Boards of Lund University and Karolinska Institute.

### DANISH TGCC PATIENTS

In the period between 1999 and 2008, DNA was collected from approximately 300 TGCC patients on the occasion of semen banking prior to surgery or fertility assessment after treatment. One hundred samples were random selected for this study; the criterion for selection being sufficient DNA amount. In total, 11 patients were excluded because of mistaken diagnosis (*n* = 6), purely extra-gonadal tumor (*n* = 4), and in one case because the genetic SNP analysis failed. Among the remaining 89 patients, 5 presented with CIS only and were therefore not included in the analysis of distribution of seminomas (SE) versus non-seminomas (NSE). For six patients no information on stage was available.

### CONTROL SUBJECTS

Control subjects were recruited in a study of reproductive function among Swedish military conscripts aged 18–20 years in the period 2000–2001 (Richthoff et al., 2002). As part of the investigation, scrotal palpation and ultrasound was performed in order to exclude testicular tumors or microcalcifications, which are indicative of an increased risk of CIS. Furthermore, they delivered one ejaculate for semen analysis as well as a blood sample for assessment of hormone levels. Among the 305 men that were eligible, 214 men with a Swedish mother were selected as control subjects (**Table 1**).

**Table 1:** Characteristics of controls and case patients.

	Control	TGCC-SE	TGCC-DK
	*n* (%)	*n*(%)	*n* (%)
*n*	214	278	89
Age (year)	18 ± 0	31 ± 7	31 ± 8
**Histology**
NSE		153 (55.0)	34 (38.2)
SE		125 (45.0)	50 (56.2)
CIS only			5 (5.6)
**Stage**
Localized		200 (71.9)	65 (73.0)
Metastasis		78 (28.1)	18 (20.2)
Unknown			6 (6.7)
**Family history of TGCC**
Yes	0 (0)	9 (3.2)	7 (2.3)
No	214 (100)	250 (90.3)	77 (88.5)
Unknown		19 (6.5)	5 (9.2)
**History of cryptorchidism**
Yes	7 (3.3)	23 (8.3)	13 (14.6)
No	207 (96.7)	247 (88.9)	63 (70.8)
Unknown		8 (2.9)	13 (14.6)

### GENOTYPING

Genomic DNA was prepared from peripheral leukocytes using QIAamp DNA Maxi Kit (Qiagen, Germany). All samples were normalized to the same DNA concentration and the genotypes were determined using the Sequenom MassArray MALDI-TOF mass spectrometry. Eleven SNPs in the gene encoding aryl hydrocarbon receptor (*AHR*) and 18 SNPs in the gene encoding aryl hydrocarbon receptor repressor (*AHRR*) with a minor allele frequency >0.05 that were identified as haplotype-tagging SNPs were selected using dbSNP (available at: http://www.ncbi.nlm.nih.gov/SNP) and SNP assays were designed using MassArray Assay Design ver. 2 software (Sequenom Inc., USA). Primers were obtained from Metabion GmbH (Germany) and all reactions were run under the same conditions, except for the primer annealing temperature of the primary PCR. PCR reactions were performed in a total volume of 6 μL containing 2.5 ng template DNA, 1.25× *Taq* PCR buffer (Hotstar, Qiagen), 0.15 U *Taq* polymerase (Hotstar, Qiagen), 3.5 nM MgCl_2_, 0.5 mM dNTP, and 100 nM of each primer. Amplifications were performed using GeneAmp 9700 machines with dual-384 heads as follows: 95°C for 15 min, 45 cycles at 95°C for 20 s, 56°C, 60°C, or 64°C for 30 s, 72°C for 60 s, and finally 72°C for 3 min. Dephosphorylation of unincorporated dNTP was achieved using shrimp alkaline phosphatase. Concentrations of individual homogenous MassEXTEND (hME) primers were adjusted to even out peak heights in the mass spectrum. The extension reactions were carried out by mixing the adjusted primer mix (containing approximately 1 μM of each primer) with hME mix containing buffer and 50 μM of each d/ddNTP mix and 1.25 U of Thermo Sequenase (Amersham Biosciences, Uppsala, Sweden). PCR amplification of hME reactions was performed as follows: 94°C for 2 min and 99 cycles at 94°C for 5 s, 52°C for 5 s, and 72°C for 5 s. The samples were then manually desalted by using 6 mg of Clean Resin and a dimple plate and subsequently transferred to a 384-well SpectroCHIP using a nanodispenser. Randomly selected samples of each genotype were directly sequenced in order to validate the SNP assays.

### STATISTICAL ANALYSIS

Single-nucleotide polymorphisms data was processed and analyzed using the web-based SNPator data analysis suite (Morcillo-Suarez et al., 2008). Agreement with Hardy–Weinberg equilibrium was tested using a χ^2^ goodness-of-fit test. TGCC patients were divided in two groups depending on whether they were diagnosed with SE or NSE. Logistic regression association was used to calculate the odds ratio (OR) and 95% confident interval for developing TGCC, for developing either SE or NSE, and for developing disseminated disease [defined by stages II, III, and IV according to the Royal Marsden Hospital staging classification (Husband and Koh, 2004), with stage I disease defined as tumor confined to the testis, with no evidence of metastases]. In the latter analysis histological subtype was included as a covariant in the logistic regression. Associations were considered statistically significant at *p* < 0.05. Assuming a co-dominant additive model, we tested a linear trend of increasing effect in the different genotypes using linear-by-linear association χ^2^ statistics (SPSS ver. 21). Linkage disequilibrium (LD; i.e., non-random association of alleles) was assessed for each gene. The LD between identified SNPs was determined by pairwise comparisons of correlation coefficient between SNPs (*r*) and the likelihood that recombination has occurred between SNPs (*D*′). Since we expected that the selected SNPs have a small contribution to TGCC and we regard our study to be exploratory rather than confirmatory, we have chosen to avoid correction for multiple testing. This may increase the risk of type 1 errors, but it prevents type 2 errors.

## RESULTS

No significant deviations from Hardy–Weinberg equilibrium were detected for the SNPs included in the analysis.

We did not find a statistically significant association between the studied polymorphisms and the risk of TGCC.

For four SNPs in *AHRR* a significant allele association with the occurrence of disseminated disease was observed (**Table 2**). Patients with metastatic disease had significantly lower frequencies of the minor rs2466287 G, rs2672725 G, rs6879758 C, and rs6896163 G alleles compared to patients with localized disease (5 vs 10%, 6 vs 11%, 2 vs 5%, and 2 vs 6%, respectively), which were associated with 57, 51, 73, and 66%, reduced per allele OR for developing metastatic TGCC, respectively. Heterozygous carriers of *AHRR* variants rs6879758 and rs6896163 had a 74 and 67% reduced risk of developing metastatic TGCC, respectively. Due to very low frequencies, or even absence, of homozygous carriers of the *AHRR* variants the OR could not be calculated for these groups of patients. However, trend analyses demonstrated significant associations for all four *AHRR* variants.

**Table 2:** Allele and genotype distributions with associated OR and trend analysis in TGCC patients stratified according to the occurrence of metastasis.

SNP (gene)	Allele and genotype	Combined				Sweden				Denmark			
		Localized	Metastasis	*P*-value	OR (95% CI)	Localized	Metastasis	*P*-value	OR (95% CI)	Localized	Metastasis	*P*-value	OR (95% CI)
		*n* (%)	*n* (%)			*n* (%)	*n* (%)			*n* (%)	*n* (%)	
rs2466287 *(AHRR)*	C	472 (90)	181 (95)		Referent	356 (90)	147 (95)		Referent	116 (89)	34 (94)		Referent
	G	54 (10)	9 (5)	0.021	0.43 (0.21–0.90)	40 (10)	7 (5)	0.036	0.42 (0.19–0.97)	14 (11)	2 (6)	0.348	0.49 (0.11–2.27)
	CC	213 (81)	86 (91)		Referent	161 (81)	70 (91)		Referent	52 (80)	16 (89)		Referent
	CG	46 (17)	9 (9)	0.061	0.49 (0.23–1.03)	34 (17)	7 (9)	0.089	0.47 (0.20–1.12)	12 (18)	2 (11)	0.026	0.098 (0.013–0.75)
	GG	4 (2)	0 (0)	–	–	3 (2)	0 (0)	–	–	1 (2)	0 (0)	–	–
	Trend			0.024				0.041				0.359	
rs2672725 *(AHRR)*	C	467 (89)	179 (94)		Referent	351 (89)	146 (95)		Referent	116 (89)	33 (92)		Referent
**	G	59 (11)	11 (6)	0.031	0.49 (0.25–0.94)	45 (11)	8 (5)	0.028	0.43 (0.20–0.93)	14 (11)	3 (8)	0.670	0.75 (0.20–2.78)
	CC	209 (79)	84 (88)		Referent	157 (79)	69 (90)		Referent	52 (80)	15 (83)		Referent
	CG	49 (19)	11 (12)	0.104	0.56 (0.28–1.13)	37 (19)	8 (10)	0.088	0.49 (0.22–1.11)	12 (18)	3 (17)	0.014	0.078 (0.010–0.60)
	GG	5 (2)	0 (0)	–	–	4 (2)	0 (0)	–	–	1 (2)	0 (0)	–	–
	Trend			0.035				0.033				0.674	
rs6879758 *(AHRR)*	G	499 (95)	187 (98)		Referent	375 (94)	152 (99)		Referent	124 (95)	35 (97)		Referent
**	C	29 (5)	3 (2)	0.025	0.27 (0.08–0.92)	23 (6)	2 (1)	0.023	0.21 (0.050–0.92)	6 (5)	1 (3)	0.627	0.59 (0.069–5.00)
	GG	235 (89)	92 (97)		Referent	176 (88)	75 (97)		Referent	59 (91)	17 (94)		Referent
	GC	29 (11)	3 (3)	0.032	0.26 (0.079–0.89)	23 (12)	2 (3)	0.034	0.20 (0.047–0.89)	6 (9)	1 (6)	0.147	0.32 (0.070–1.49)
	CC	0 (0)	0 (0)	–	–	0 (0)	0 (0)	–	–	0 (0)	0 (0)	–	–
	Trend			0.022				0.020				0.622	
rs6896163 *(AHRR)*	A	497 (94)	188 (98)		Referent	373(94)	153 (98)		Referent	124 (95)	35 (97)		Referent
**	G	31 (6)	4 (2)	0.037	0.34 (0.12–0.98)	25 (6)	3 (2)	0.035	0.29 (0.087–0.98)	6 (5)	1 (3)	0.627	0.59 (0.069–5.00)
	AA	233 (88)	92 (96)		Referent	174 (87)	75 (96)		Referent	59 (59)	17 (94)		Referent
	AG	31 (12)	4 (4)	0.040	0.33 (0.11–0.95)	25 (13)	3 (4)	0.041	0.28 (0.082–0.95)	6 (6)	1 (6)	0.180	0.41 (0.11–1.50)
	GG	0 (0)	0 (0)	–	–	0 (0)	0 (0)	–	–	0 (0)	0 (0)	–	–
	Trend			0.032				0.031				0.622	

Polymorphisms in *AHRR* did not show a significant association with risk of TGCC or histological subtype, which was also the case for SNPs in *AHR*. Histological subtype and the occurrence of metastasic disease were not associated with SNPs in *AHR*. LD analysis demonstrated a high correlation between rs2466287 and rs2672725 as well as between rs6879758 and rs6896163 (**Table 3**).

**Table 3:** Linkage disequilibrium (*D*′) and correlation coefficients (*r*) between SNPs in *AHRR* that associate with metastatic TGCC in the total population.

	rs2466287		rs2672725		rs6879758	
	*D*′	*r*	*D*′	*r*	*D*′	*r*
rs2466287						
rs2672725	0.978	0.909				
rs6879758	0.239	0.172	0.230	0.155		
rs6896163	0.195	0.142	0.186	0.132	0.999	0.948

When analyzed in the two populations individually, similar statistically significant ORs for the four SNPs were observed in the Swedish population as compared to the combined population. The associations in the Danish populations showed similar tendencies, although these did not reach statistical significance.

## DISCUSSION

In this study we have analyzed associations between SNPs in genes encoding AHR and AHRR and the risk of developing TGCC, histological subtype, and the occurrence of metastasis. Whereas no associations were found with SNPs in *AHR*, four variants in *AHRR* associated significantly with the occurrence of metastatic disease.

Both AHR and its negative regulator AHRR are ligand-activated transcription factors that belong to the family of basic helix-loop-helix Per-Arnt-Sim (bHLH-PAS) proteins. They are ubiquitously expressed in almost all human tissues, with levels particularly high in the testis (Burbach et al., 1992; Tsuchiya et al., 2003). 2,3,7,8-Tetrachlorodibenzo-*p*-dioxin (TCDD) is the most well-characterized exogenous ligand of AHR and it is known for its anti-estrogenic properties such as inhibition of estradiol-induced uterine weight increase and decreased levels of estrogen and progesterone receptors in uterus of rat and mice (Safe, 1995).

Several epidemiological studies have shown that TCDD promotes cancer, and experiments in AHR-deficient mice have shown that AHR is essential for the tumor-promoting effects of TCDD (Andersson et al., 2002; McMillan and Bradfield, 2007). Conversely, transgenic mice with a constitutively active AHR spontaneously develop tumors, their weights of testis and ventral prostate are decreased and the epididymal sperm reserve is reduced (Brunnberg et al., 2011). Furthermore, AHRR, the negative regulator of AHR signaling, functions as a tumor suppressor in multiple human tumors (Zudaire et al., 2008; Li et al., 2012). Several studies have shown that the AHR activation also interferes with reproductive health. An increased incidence of TGCC has been observed in workers exposed to TCDD (Kogevinas et al., 1997) and dioxins have been reported to reduce sperm number, and to decrease accessory sex gland weight and anogenital distance in rats (Mably et al., 1992; Gray et al., 1995; Sommer et al., 1996; Faqi et al., 1998; Ohsako et al., 2002).

While SNPs in either AHR or AHRR did not associate with the risk of TGCC, it is interesting that an association was observed between AHRR and dissemination of TGCC, since AHR signaling is indeed known to contribute to the control of cell adhesion and migration (Carvajal-Gonzalez et al., 2009; Dietrich and Kaina, 2010). A potential role of AHR signaling in dissemination of TGCC is further supported by Koliopanos et al. (2002) who reported that AHR agonists decreased anchorage-independent pancreatic cancer cell growth.

Since testicular cancer rarely occurs before the onset of puberty, the malignant transformation of CIS cells may be associated with activation of the hypothalamic–pituitary–gonadal axis. An interaction between AHR signaling and steroid or gonadotropic hormones could therefore be an alternative mechanism underlying the association between AHRR polymorphisms and metastatic TGCC.

A strength of our study is the access to two independent TGCC populations. Although not all findings reached statistical significance in the Danish cohort when the countries were analyzed separately, most probably due to the limited number of cases in this group, the same trends with similar relative risks were found, which strengthens the relevance of our findings. We cannot exclude that a small fraction of the TGCC patients in our cohort were from non-Caucasian background. However, none of the allele frequencies of the analysed SNPs deviated from Hardy-Weinberg equilibrium, which indicates that either the non-Caucasian allele frequencies did not differ from those in the Caucasian population, or that non-Caucasians did not significantly contribute to the total cohort. The SNPs identified in this study are intronic polymorphisms, thus excluding structural changes in the proteins that these genes encode. Yet, polymorphisms in non-coding regions can still play an important role in the regulation of gene expression, e.g., by affecting mRNA stability, regulation by intronic micro RNAs, or potential splice sites. The identified SNPs could also be in LD with other genetic polymorphisms that are causally related to TGCC.

In summary, we have identified polymorphic variants in the gene encoding AHRR that associated with risk of disseminated TGCC. This supports our hypothesis that the AHR signaling pathway may affect the progression of TGCC, possibly by interfering with reproductive hormone actions.

## Conflict of Interest Statement

The authors declare that the research was conducted in the absence of any commercial or financial relationships that could be construed as a potential conflict of interest.
